# Progress in the Synthesis Process and Electrocatalytic Application of MXene Materials

**DOI:** 10.3390/ma16206816

**Published:** 2023-10-23

**Authors:** Peng Wang, Bingquan Wang, Rui Wang

**Affiliations:** 1 School of Environmental Science and Engineering, Shandong University, Qingdao 266237, China; 2 School of Chemistry and Molecular Engineering, Qingdao University of Science and Technology, Qingdao 266042, China

**Keywords:** MXene, 2D materials, electrocatalysis, MXenes composites

## Abstract

With their rich surface chemistry, high electrical conductivity, variable bandgap, and thermal stability, 2D materials have been developed for effective electrochemical energy conversion systems over the past decade. Due to the diversity brought about by the use of transition metals and C/N pairings, the 2D material MXene has found excellent applications in many fields. Among the various applications, many breakthroughs have been made in electrocatalytic applications. Nevertheless, related studies on topics such as the factors affecting the material properties and safer and greener preparation methods have not been reported in detail. Therefore, in this paper, we review the relevant preparation methods of MXene and the safer, more environmentally friendly preparation techniques in detail, and summarize the progress of research on MXene-based materials as highly efficient electrocatalysts in the electrocatalytic field of hydrogen precipitation reaction, nitrogen reduction reaction, oxygen precipitation reaction, oxygen reduction reaction, and carbon dioxide reduction reaction. We also discuss the technology related to MXene materials for hydrogen storage. The main challenges and opportunities for MXene-based materials, which constitute a platform for next-generation electrocatalysis in basic research and practical applications, are highlighted. This paper aims to promote the further development of MXenes and related materials for electrocatalytic applications.

## 1. Introduction

**Figure 1:**
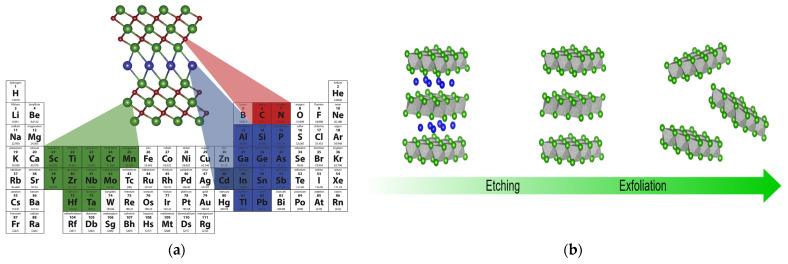
(**a**) Chemical composition of MAX [10]. (**b**) Processes for MAX etching to create multilayer MXene and stripping to create monolayer MXene [11].

MXenes are a class of two-dimensional materials obtained through the selective etching of A-layer atoms by using MAX precursors. The first MXene member, Ti_3_C_2_T_x_, was prepared by Prof. Yury Gogotsi, Prof. Michel Barsoum, and PhD student Michael Naguib at Drexel University in 2011 [1]. The chemical composition of MAX and its processes of etching to form multilayer MXene and peeling to form monolayer MXene are shown in Figure 1. It is discovered that MXenes have greater specific surface area than their parent substances [2]. M is an early transition metal (Ti, V, Cr, etc.) and X is carbon or nitrogen or carbon nitride [3]. An MXene has a structure of *n* + 1 M intermediate layers and *n* X layers; the more common ones are M_2_X, M_3_X_2_, M_4_X_3_, and M_5_X_4_. The types of maternal MAX are very rich, and more than 100 types have been identified, so there are many more types of MXenes that can be fully developed [4]. Simultaneously, the surfaces of MXene functional groups greatly improve their properties. MXenes have a hexagonal symmetric lattice [5] wherein X is located in the octahedral gap formed by M. Wet chemistry produces MXenes in the form of colloidal solutions, which can be made in a variety of colors through dilution. Materials made of MXenes have a significant specific surface area and active surface characteristics. In addition, MXenes also offer good mechanical strength and toughness due to their strong covalent connections and a certain thickness of crystal atoms [6]. A functional group is created on the surfaces of MXenes during the etching process, which decreases the chemical potential energy of the compound and gives it outstanding features like some resistance to oxidation and thermal endurance [7]. Nevertheless, because of the high van der Waals interactions between an MXene’s layers, it has a strong aggregation ability, and the effective surface area is reduced as a result [8]. The solution to overcome this problem is to use MXenes as a substrate layer. External components (embedding or spacing) are introduced between the nanosheets and assembled through cross-linking (gelation) or templating to form 3D and porous structures [9]. Currently, MXenes are mainly studied as carbide MXenes rather than nitride MXenes. This is because the A-layer of n-based MAX is more challenging to peel than c-based MAX, and the structural stability of nitride MXenes is poor for long-term preservation.

The synthesized MXene-based materials are widely used in alkali metal batteries, supercapacitors, membrane separations, etc. and have been showing excellent performance. The excellent electrical conductivity and hydrophilicity of MXenes also ensure their stability as electrocatalysts in the hydrogen evolution reaction (HER) [12], oxygen evolution reaction (OER) [13], oxygen reduction reactions (ORR) [14], nitrogen reduction reactions (NRRs) and CO_2_ reduction reactions (CO_2_RR) [15,16]. However, studies on the application of such materials in electrocatalysis are scarce and mainly limited to Ti_3_C_2_T_x_ and Mo_2_CT_x_.

It is undeniable that although MXenes were discovered more than a decade ago, research on them is still in the exploratory stage and there is a large untapped potential waiting to be realized. In recent electrocatalytic studies of MXenes, the mixture of MXenes with monoatomic transition metals has been found to be a very valid modification method, and more research is being conducted to address this approach. For example, Zhang et al. prepared Mo_2_TiC_2_T_x_-Pt_SA_ as an HER catalyst. Due to the strong Pt-C covalent bond between Pt atoms and Mo_2_TiC_2_T_x_, electrons are transferred from Pt to Mo_2_TiC_2_T_x_, preventing the aggregation of single Pt atoms. The charge-transfer resistance is much lower than that of Mo_2_TiC_2_T_x_, and the stability and catalytic activity of Mo_2_TiC_2_T_x_-Pt_SA_ are significantly improved [17]. In the past five years, the Mxene family has expanded to more than 30 members with promising applications in many fields due to the diversity resulting from the combination of transition metals with C/N. With the combination of metallic conductivity and hydrophilicity, MXenes have demonstrated their potential in a wide range of applications, such as in electrochemical energy storage (e.g., batteries and supercapacitors), biomedical applications, sensor technology, and electromagnetic shielding [18]. At the same time, MXene materials have great development potential in the field of hydrogen storage, so MXene materials are expected to become a promising alternative material to enhance hydrogen storage capacity. Therefore, it is necessary to keep up to date with the latest research advances related to the synthesis of MXene materials and their electrocatalytic applications, as well as to address the current obstacles in this field. There are various reviews on the preparation, properties, and applications of MXenes in energy storage and conversion, as shown in Table 1; however, to the best of our knowledge, a comprehensive description and evaluation of the latest results on MXene-based electrocatalytic materials remains a gap in the knowledge base. In addition, there are few reviews focusing on MXene-based electrocatalyst materials. In this paper, we provide a comprehensive review of MXene materials from an electrocatalytic perspective. We first discuss synthesis methods such as HF etching and its derivatives, electrochemical etching and molten salt etching, and then focus on recent advances in electrocatalysis.

## 2. The Principal Techniques for Producing MXene Materials

MXenes are commonly synthesized by selectively etching particular atomic layers from layered precursors such as MAX phase materials. The initial MXene was synthesized using room-temperature HF etching, and subsequent types of MXenes, including TiC_2_T_x_, Mo_2_CT_x_, V_2_CT_x_, etc., have been successfully obtained using this approach [28,29]. Nevertheless, HF aqueous etching requires handling high concentrations of HF and goes through a complex multi-step process. Since the prepared MXenes have poor stability in long-term storage and the reagents used in this process can be more harmful to the environment, researchers started investigating some milder and more environmentally friendly preparation methods. This led to the development of green methods such as alkali treatment, electrochemical etching, and molten salt etching. Ghidiu et al. developed an easy-to-operate and safer method for the synthesis of MXenes, which generates HF in situ through the reaction of HCl and LiF [30]. Afterwards, other fuel compounds, such as NaF, KF, FeF_3_ and tetrabutylammonium fuel compounds, were also used to synthesize MXenes [31,32]. Although various MXenes have been obtained via etching and its derivative methods, because of the acute toxicity of HF, these approaches severely limit the synthesis and application of MXene-based catalysts on a broad scale, so we urgently need to develop new HF-free synthesis methods. Gabrijela Ljubek et al. prepared MXense Ti_3_C_2_T_x_ at room temperature through the mechanochemical ball milling of Ti_3_AlC_2_ using different salts [33]. In another study, Li and coworkers discovered that in the presence of a small amount of water, KOH can act as an etching agent in the preparation of MXenes. Xiu et al. prepared MXenes by modulating MXene in molten salt systems and using Lewis acid diversity and green chemistry to provide a safer and efficient method for MXene preparation [34]. Li et al. reported a method of elemental replacement via reaction with molten salts [35]. It can be seen from the above that the future development trend of MXenes may focus on safe and efficient preparation methods.

### 2.1. Etching of Synthetic MXene Materials and Methods for Their Derivation

#### 2.1.1. Etching with Hydrofluoric Acid (HF)

**Figure 2:**
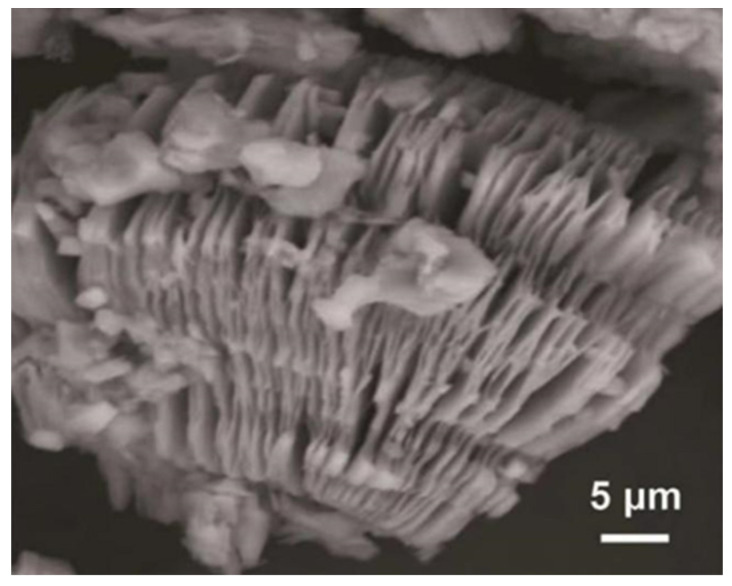
Accordion-like Ti_3_C_2_T_x_ is produced by reaction of deionized water with hydrofluoric acid solution [1].

Etching methods have been widely employed in MXene research; however, hydrofluoric acid etching is still the most regularly used approach. The main idea behind this approach is that F combines with A-layer atoms in MAX, and the A-layer atoms are removed from MAX to produce layered MXenes, resulting in the generation of hydrogen. In 2011, Naguib et al. demonstrated a method for producing Ti_3_AlC_2_ MAX phase by using HF acid etching [1]. The method used a simple replacement process to remove the Al layer from the Ti_3_AlC_2_ MAX phase by using HF acid, which then generated H_2_. In addition, deionized water reacted with hydrofluoric acid solution to generate Ti_3_C_2_T_x_ (where T stands for -O, -F, and -OH) and H_2_, and the Ti_3_C_2_T_x_ was obtained in the form of an accordion [1]. The accordion-like Ti_3_C_2_T_x_ is shown in Figure 2. Afterwards, Naguib et al. successfully prepared a series of MAX complexes such as Ti_2_AlC, (Ti_0.5_Nb_0.5_)_2_AlC, Ti_3_AlCN, Ta_4_AlC_3_, (V_0.5_Cr_0.5_)_3_AlC_2_, and Nb_2_AlC, as well as Zr_3_Al_3_C_5_, Ti_3_SiC_2_, Mo_2_Ga_2_C, etc., by using HF acid etching to exfoliate MXenes [36]. HF acid etching has long been the most practical and popular process for preparing MXene compounds. In the HF acid etching process, the concentration of the etchant, reaction time, and temperature play very critical roles in designing high-quality MXene layers. For MAX phases with large n values, it is usually necessary to use a higher concentration of hydrofluoric acid for etching. Nevertheless, if the MAX phase with a low n value is etched with a stronger hydrofluoric acid solution, it may lead to over-etching, resulting in the dissolution of the generated MXene. Therefore, in order to obtain the complete MXene product, it is required to use a low concentration of HF acid for etching the MAX phase with a small n. Kim et al. studied the etching process of the maximal phase, Ti_3_AlC_2_, under different etching conditions on an atomic scale using an ion beam and an electron microscope [37]. They studied the change in Ti_3_AlC_2_ phase structure by constructing the correlation function between the etching agent and the etching time and found that although it interacts with the HF etching agent, the Al atom at the edge of the middle layer of the largest phase Ti_3_AlC_2_ does not participate in the etching [38]. After a period of time, researchers began to realize that there were many shortcomings in MXenes obtained by using HF etching, and at the same time, there were certain risks in this method, which led to a gradual decrease in the study of related methods. Nevertheless, the HF acid etching method opened up the study of MXenes and had a key influence on the late preparation of MXenes and the fluorine-free study of MXenes.

#### 2.1.2. Modified Etching Method for Synthesizing MXene Materials

Because the acid fluoride solution is corrosive and toxic, the process of extracting Al layer directly from the MAX phase by using HF acid is dangerous, and the relevant personnel are studying ways to avoid this situation. Replacing HF acid with fluoride salts (e.g., LiF, NH_4_HF_2_, FeF_3_, KF, and NaF) and HCl is currently the most common method, i.e., in situ HF acid etching. Typically, in the process of synthesizing MXenes by etching the MAX phase with HF acid, a byproduct (AlF_3_·3H_2_O) is formed, and researchers have devised a modified etching method in order to synthesize MXenes without this impurity. This method was first reported by Halim et al., who successfully grew Ti_3_C_2_ film on Ti_3_AlC_2_ film by using sputtering deposition technology, which provided help for MXene etching [39]. Subsequently, Ti_3_C_2_T_x_ powder was effectively etched by Feng et al. using a 1 M NH_4_HF_2_ aqueous solution. The ideal reaction time for this technique was 8 h at 60 °C [40,41]. And, meanwhile, the study showed that compared with Ti_3_C_2_T_x_ obtained by using HF etching, the method used to Ti_3_C_2_T_x_ obtained by this method was shown to have better thermal stability as well as structural integrity compared to Ti_3_C_2_T_x_ obtained by using HF etching [42]. The researchers also tried to extend the etching agent to the application of NaHF_2_ and KHF_2_ and found that these two etchers achieved better results in the preparation of MXenes. Etching with NaHF_2_ and KHF_2_ provides better etching efficiency and control, contributing to high-quality MXene materials. These new etchants expand the range of options for MXene synthesis and offer more possibilities for the further optimization of the preparation process. Cockreham et al. derived the conditions leading to the formation of the byproduct AlF_3_·3H_2_O during the etching process with cobalt fluoride (i.e., CoF_2_/CoF_3_) [43]. SEM micrographs of the CoF_3_/MAX samples did not show any AlF_3_·3H_2_O impurities. During the acid etching process, cations could be inserted into the gaps in the MXene’s layered structure, resulting in greater distances between layers, which minimized the internal forces between the layers and possibly delaminated the material layers during sonication. This increase in interlayer distance may have an impact on the structure and properties of MXenes, such as their conductivity, mechanical properties, etc. This technique streamlines the multi-step synthesis procedure and makes it possible to synthesize several MXene layers in a single step.

#### 2.1.3. Etching Using Modified Fluoride-Based Acid

To avoid the toxic effects of the HF etching process, researchers have been trying to find preferable ways to remove the atomic layers of MAX. In addition to direct HF solutions, the etching process can also employ mixtures of fluoride salts (e.g., KF, NaF, and NH_4_F) with strong acids. After the selective etching of atoms by using fluoride salts and strong acids, cations are embedded in situ (e.g., K^+^, Na^+^, and NH_4_^+^) [38]. When water is inserted in between the MXene layers, the distance between them expands and the contact between the layers decreases. This may affect the structure and properties of MXene materials. However, it is important to note that the content of fluoride salts and strong acids used in the MXene synthesis process ultimately affects the quality and size of the MXene material produced. In order to solve this problem, ultrasonic methods need to be utilized. For example, multilayered Ti_3_C_2_ is prepared by using the clay method, i.e., it is subjected to a sonication step that delaminates it into monolithic flakes, but this tends to leave the MXene flakes with small defects [44].

#### 2.1.4. Molten Salt Etching Synthesis of MXene

**Figure 3:**
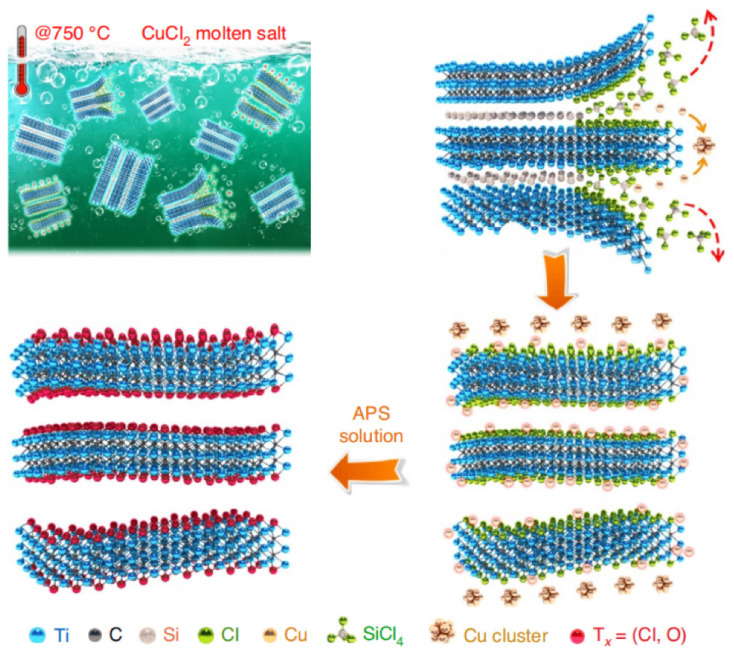
Accordion-like MXene was prepared using Ti_3_SiC_2_ and CuCl_2_ at 750 °C. The red dashed arrows represent reaction (1); The solid orange arrows represent reaction (2) [46].

Molten salt etching is a non-HF etching method for synthesizing MXenes that avoids the safety hazards associated with the use of HF. In this method, an MXene is synthesized by heating the MAX phase (e.g., Ti_4_AlN_3_) in a mixture of molten fluoride salts in an argon-shielded environment at 550 °C, and the etching process is typically completed in less than 30 min. The molten salt etching method’s comparatively quick processing time is one of its benefits. It uses the molten salt’s anions as the etchant; the anions are equivalent to F^−^ in HF while the cations are equivalent to H^+^ in HF [8]. The molten salt’s anions interact with the MAX’s A layer during the etching procedure to produce MXenes. However, only a few particular types of MXene materials have been successfully synthesized using the molten salt etching technique. Considering that carbide MXenes are more stable than nitride MXenes, their formation requires higher energy. As a result, applying the etching procedure described above to produce nitride MXene materials is challenging. Some researchers have developed new methods to synthesize nitride MXenes. For example, Urbankowski et al. successfully synthesized the first nitride MXene, Ti_4_N_3_, using a mixture of KF, LiF, and NaF melted at 550 °C in an Ar environment for 30 min [45]. Similarly, Li et al. used a ZnCl_2_ Lewis acid molten salt etched at 550 °C to Ti_3_AlC_2_ to synthesize Ti_3_ZnC_2_ [35]. Li et al. were inspired by this and created an MXene that resembled an accordion using Ti_3_SiC_2_ and CuCl_2_ at 750 °C [46]. The accordion-like MXene prepared by Li et al. using Ti_3_SiC_2_ and CuCl_2_ at 750 °C is shown in Figure 3. 

To accomplish the etching, they employed a direct redox connection between the Lewis acid molten salt cation and element A in MAX. This method shows how altering the type of Lewis anion can alter the surface chemistry of MXene materials and the surface functional groups. It should be mentioned that to eliminate the metal byproducts and create -O functional groups on their surfaces, MXene materials produced by molten salt etching need to be submerged in ammonium persulfate (APS, (NH_4_)_2_S_2_O_8_) solution [8]. However, the MXene materials produced by this process are not as hydrophilic as HF MXene because they lack -OH functional groups.

#### 2.1.5. Electrochemical Etching Method

**Figure 4:**
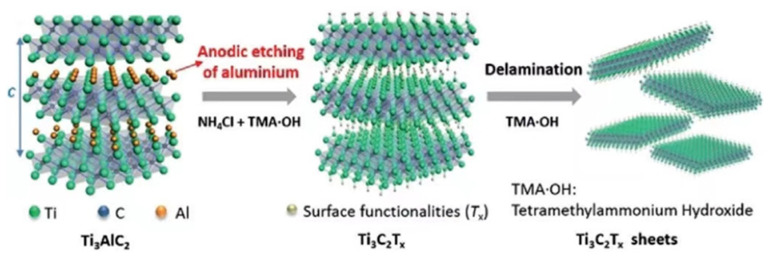
Etching and delamination processes [47].

Electrochemical etching is a method that enables the efficient etching of MAX precursors and the selective extraction of layered nanomaterials. Yang et al. showed an efficient electrochemical etching route for the preparation of Ti_3_C_2_T_x_ MXene (T = O, OH) as shown in Figure 4 [47]. The etching procedure was carried out in a standard two-electrode setup. For the etching method, two blocks of Ti_3_AlC_2_ were employed as the working electrode (anode) and counter electrode (cathode), and the aqueous electrolyte included 1.0 M NH_4_Cl and 0.2 M tetrame-thylammonium hydroxide (TMAOH). In this study, chloride ions accelerated the etching of Al and the breaking of TieAl bonds, and NH_4_OH exhibited a significant embedding effect at the edge of the etched anode [48]. This composite electrolyte aided in etching the undersurface and allowed 2D MXene to be successfully stripped from the block. Similarly, Sun et al. showed the feasibility of electrochemically etching the Ti_2_AlC phase in dilute hydrochloric acid to obtain 2D Ti_2_CT_x_ MXene [49]. In addition, since electrochemical etching does not use HF, the products are more hydrophilic relative to HF MXene because they do not have fluorine termini. The electrochemical etching technique offers more potent energy, breaks through the etching energy barrier, increases the variety of etching conditions and compositions, and permits a larger variety of etching systems. This method offers the possibility of achieving more precise controllability in 2D MXene surfaces. It should be noted that different electrochemical etching methods may require the selection of different electrolyte systems and operating conditions to obtain specific types of MXene materials.

### 2.2. Hydrothermal Synthesis

**Figure 5:**
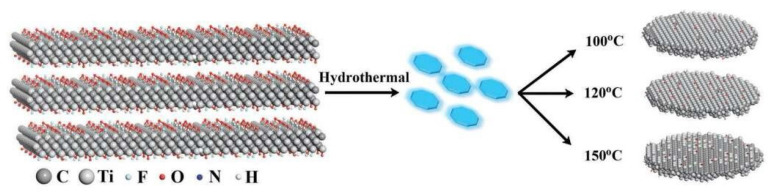
Preparation of water-soluble Ti_3_C_2_ MXene by hydrothermal technology [50].

This is a non-homogeneous reaction that involves heating an aqueous solution above the boiling point of water in an autoclave containing the precursor material. In this reaction, the synergistic effects of temperature, pressure, and solution pH affect the size, shape, structure, and properties of the quantum dots. Meanwhile, the solution pH, reaction temperature, and reaction time also play crucial roles in the generation of MXenes. By modulating these parameters, the controlled synthesis of MXene materials can be realized. Xue et al. successfully prepared water-soluble Ti_3_C_2_ MXene using the hydrothermal technique and found that by adjusting the hydrothermal reaction temperature to 79 °C, they could tailor the nature, thickness, and size of the MXene produced [50]. In Figure 5, the synthesis process is depicted. In the experiments conducted at different temperatures, they obtained particles with average diameters of 2.9, 3.7, and 6.2 nm and average thicknesses of 0.99, 0.91, and 0.89 nm, respectively. These results indicate that the particles are mainly composed of monomolecular layers. The researchers also observed that during the reaction, the Ti_3_C_2_ quantum dots exhibited surfaces containing -NH functional groups, new MXene structures were formed at low-temperature conditions (e.g., 100 °C), and d-spacing values could be determined. Moreover, in the MXene formed at 120 °C, the core structure was CTi and the surface structure was a fusion structure of TiO_2_. However, under high temperature conditions (i.e., 150 °C), most of the Ti atoms were etched away, leading to the formation of amorphous MXene structures. The choice of reaction temperature and time, on the other hand, affects the reaction rate and the crystal structure of the products. By optimizing the non-homogeneous reaction conditions, the specific morphology, size, and properties of the desired MXene material can be obtained. This method provides an efficient way to synthesize MXene materials with specific properties. The hydrothermal technique is an environmentally friendly and successful way to produce a wide range of 2D MXenes with different compositions and to avoid contact with highly toxic HF vapors. Cai et al. immersed Ti_3_AlC_2_ in an aqueous NaOH solution at about 85 °C for 100 h, followed by a hydrothermal treatment using 1 M sulfuric acid for 1.5 h at 85 °C [51]. This method is used for the production of Ti_3_C_2_T_x_ in the MAX system. By using this method, the effective removal of the aluminum layer can be achieved to form Ti_3_C_2_T_x_ MXene. Another method is to soak Ti_3_AlC_2_ fine powder in an aqueous ammonium fluoride solution and then subject the mixture to a hydrothermal chemical reaction [52]. Similar methods have been used to prepare other MXene materials such as Nb_2_C and Ti_3_C_2_ using a hydrothermal etching reaction with sodium boron fluoride and hydrochloric acid [53]. Compared to Ti_3_C_2_ prepared by using conventional etching (using HF), Ti_3_C_2_ prepared using the hydrothermal method has the following advantages: the elimination of the aluminum layer, an enlarged lattice parameter, width of the interlayer of the 2D MXenes is maximum, and a BET surface area distribution is better. Because of the hydrothermal reaction’s gradual release mechanism, the Ti_3_C_2_ after ultrasonication is easy to dislodge, which is conducive to obtaining excellent adsorption effects. By combining the Bayer method and base-induced hydrothermal technique, it is also possible to achieve the high refinement of multilayer Ti_3_C_2_T_x_ with zero termination of embedded fluoride ions with 90% purity [54]. In addition to the hydrothermal method, there are other methods that have been recommended as effective ways to prepare MXene without involving HF such as chemical vapor deposition, salt template methods, and so on [55]. These effective and feasible modern processes are considered necessary mechanisms for the synthesis of MXene-based adsorbent materials with proper surface functional group control, maximum specific surface area, and well-defined chemical stability, which can solve many environmental problems [56].

### 2.3. Physically Assisted Synthesis of MXene

**Figure 6:**
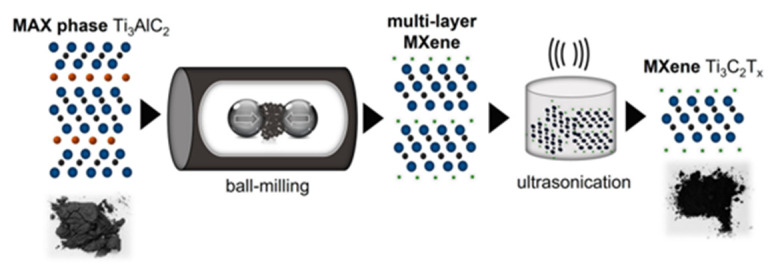
Synthesis of MXene obtained by using ball milling and ultrasonic treatment [33].

Although the techniques described above have been proved to be successful for MXene production, the further improvement and optimization of parameters such as the thickness and size of the flakes are needed for the development of interdisciplinary applications. Xue et al. reported a method for the synthesis of fluorine-free Ti_3_C_2_ MXene using a chemical combination ball milling method with a hierarchical and porous structure and an HF-treated Ti_3_C_2_ MXene with a specific surface area that was higher than the convention by a factor of 8 (38.93 m^2^ g^−1^ and 4.87 m^2^ g^−1^, respectively) [57]. Compared to other methods using tetramethylammonium hydroxide (TMAOH) and LiCl solvent and Ti_3_AlC_2_ powder, the ball milling method is a simpler and more environmentally friendly way to prepare MXenes [57]. The delamination and preparation of MXenes are achieved by the ball milling method, which mixes and grinds the raw material powder by using mechanical force. Compared with other methods, the ball milling method has fewer operating steps and does not require the use of toxic solvents or chemical reagents. In addition, there are other safer ways to prepare MXenes such as sputtering and chemical vapor deposition (CVD). Chen et al. successfully synthesized scandium-based MXene (Sc_2_CO_x_) through magnetron sputtering [58]. To synthesize the semiconductor Sc_2_CO_x_, they deposited C and Sc on silicon and sapphire substrates at room temperature with a base pressure of 6 × 10^−4^ mTorr and deposition rates of 30 and 60 nm h^−1^ for C and Sc, respectively [58]. Xu et al. synthesized a two-dimensional ultrathin metal carbide by using CVD on Cu/Mo bilayer substrate (Mo_2_C) MXene superconducting crystals with a thickness of about 100 μm [59,60]. On graphene substrates, the thickness of Mo_2_C MXene can be increased to the centimeter scale, and high quality synthesis has been observed after using Mo-Cu alloy catalysts [61]. Furthermore, Ljubek et al. prepared MXense Ti_3_C_2_T_x_ through the mechanochemical ball milling of Ti_3_AlC_2_ using different salts at room temperature [33]. This process is shown in Figure 6. The resulting material was then sonicated in hydrochloric acid or ethanol to complete the delamination process. Recently, Mei et al. proposed to selectively remove the S layer from the Ti_2_SC MAX phase in a gas stream with an argon/hydrogen (95/5) volume ratio at an optimum temperature of 800 °C and obtained 2D Ti_2_C MXene powders terminated with -O and -OH functional groups through sonication in acetone [62]. Nevertheless, although many syntheses of MAX phase and MXenes have been reported, there is still a need to find simple, environmentally friendly, and economically viable methods in order to achieve commercial-scale production.

Currently, most synthesized MXenes constitute multilayer two-dimensional structures with small layer spacings. However, in order to obtain MXene materials with fewer or single layers with increased and tunable interlayer spacing, further layer exfoliation has become necessary, which is the current hotspot and challenge for researching MXene as an electrode material application area. Currently, the most commonly used method to prepare an MXene is to strip the MAX phase through chemical etching utilizing high concentrations of HF or mixtures of LiF and HCl. However, it should be noted that HF and HCl are highly corrosive and toxic, and there is a greater danger during the experimental operation. Therefore, the currently available methods for synthesizing MXenes mainly have the following drawbacks: the etching solution is highly toxic and highly corrosive, which is dangerous and not environmentally friendly; the synthesis process is slow, usually takes a long time, and is difficult to control, and the cost is high; the synthesized MXenes usually contain end-groups such as F^−^, which adversely affects the performance of an MXene as an electrode and its composite materials. In order to achieve large-scale production, it is an important research direction to find a safe, environmentally friendly, efficient, and inexpensive preparation method to obtain large-scale MXene materials with controllable numbers of layers, excellent quality, porosity, and monolithic structure. Many safer and more environmentally friendly methods have been proposed for the preparation of MXenes, which bring hope for solving these challenges.

## 3. The Applications of MXene-Based Materials

### 3.1. Electrocatalysts for the HER Based on MXene

Nowadays, mankind is gradually shifting its dependence on fossil energy sources to clean energy sources to meet the needs of life. Because of its high energy density and environmental friendliness, hydrogen (H_2_) is seen as a prospective energy carrier as a clean energy alternative to fossil fuels. In renewable energy systems, electrochemical water decomposition (EWD) technology plays an important role, especially because it can be combined with renewable energy sources. Through a clean and sustainable method, electrochemical water breakdown via the HER enables the production of green hydrogen. The HER, as an electrochemical water-oxidation half-reaction, is a multi-step reaction process that involves steps such as hydrogen adsorption, hydrogen reduction, and H_2_ desorption [63].

#### 3.1.1. Acidic Solution HER Electrocatalysts

Electrochemical water decomposition is a promising green cathodic hydrogen production method. The method mainly relies on metal catalysts (e.g., Pt, Pd, Ru, Ir, etc.), metal nitrides, metal selenides, or phosphides (e.g., Cu, Ni, Fe, MoS_2_, Ni_2_P, etc.) to drive the hydrogen precipitation reaction. However, these metal-based catalysts are expensive, resource-limited, and cannot be mass-produced, thus limiting their widespread application [64]. In contrast, porous graphitic carbon nitride (g-C_3_N_4_) has a highly controllable thermal stability and surface-area-to-volume ratio, and can be prepared from inexpensive and common precursors (e.g., urea, melamine, thiourea, and cyanamide) via green methods for sustainable development and large-scale demand. Therefore, a relatively green, simple, and one-pot approach has been developed to synthesize various one-dimensional g-C_3_N_4_ nanostructures doped with bimetallic atoms such as PtPd/g-C_3_N_4_ nanorods, Pd/Cu/g-C_3_N_4_ nanowires, and so on [65]. Pt-based catalysts show excellent performance in the HER, but their high cost and scarcity severely limit their practical application. Therefore, it has become crucial to search for reserve-rich electrocatalysts that can replace Pt. As mentioned earlier, MXenes exhibit excellent electronic properties due to their unique metallic properties [66]. Therefore, the research on and development of MXene-based HER electrocatalysts have attracted extensive attention.

**Figure 7:**
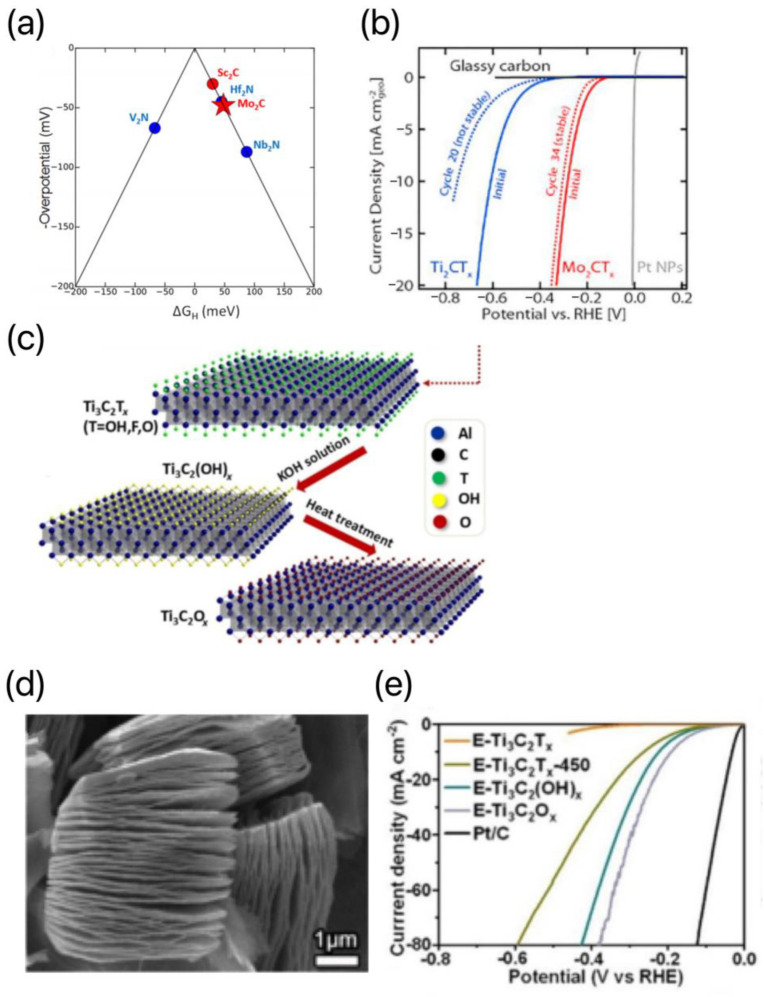
(**a**) Volcano map constructed from ΔG_H_ values. (**b**) LSV curves for acidic HER of Mo_2_CT_x_ and Ti_2_CT_x_ [67]. (**c**) Schematic diagram of the preparation of E-Ti_3_C_2_O_x_. (**d**) SEM image of Ti_3_AlC_2_. (**e**) Corresponding Tafel slopes [71].

Among all the reported MXene-based materials, Mo_2_CT_x_ exhibits the most excellent activity for an acidic HER. Seh et al. investigated, for the first time, in a comprehensive manner, Mo_2_CT_x_ MXenes as acidic HER electrocatalysts, combining theoretical calculations and experimental results [67]. They synthesized Mo_2_CT_x_ MXenes with Ga atoms removed using the HF etching of Mo_2_Ga_2_C. DFT calculations were used as ideal tools to evaluate the HER electrocatalytic potential of MXenes as they provide general rules for the qualitative prediction of HER performance. By comparing the free energy changes (ΔG_H_) before and after hydrogen adsorption on the surfaces of different materials, the HER performance of the materials can be accurately predicted [68]. They constructed a volcano diagram to aid in material screening based on ΔG_H_ values calculated by MXenes, as shown in Figure 7a. MXenes near the top of the volcano diagram have better HER properties, with good theoretical overpotentials for Sc_2_C, Hf_2_N, and Mo_2_C. In agreement with theoretical calculations, Mo_2_CT_x_ showed higher HER activity than Ti_2_CT_x_ in experimental measurements. At an overpotential of 283 mV (η_10_ = 283 mV), the synthesized Mo_2_CT_x_ MXenes reached a current density of 10 mA cm^−2^, whereas the synthesized Ti_2_CT_x_ MXenes had a current density of 609 mV under the same conditions. Figure 7b demonstrates that the HER activity of Ti_2_CT_x_ further decreases with an increase in the number of cycles, whereas the Mo_2_CT_x_ maintains a good HER activity. As a molybdenum-containing MXene, the superior electrocatalytic performance of Mo_2_CT_x_ stems from the presence of catalytically active sites on its basal plane, whereas the catalytically active sites of MoS_2_ exist only at the edges of the two-dimensional crystal structure [69]. However, the intrinsic HER performance of MXenes is still far from satisfactory and thus cannot completely replace Pt-based materials. However, the HER activity of MXenes can be further optimized by tuning the terminal T_x_, doping other transition metals and/or nonmetallic elements, and constructing surface defects.

Most MXenes are metalloconductive and hydrophilic due to the presence of terminal Tx on the MXene surface. Currently, the functionalization of MXenes with O, OH, or F has been extensively investigated via DFT calculations, and an MXene functionalized with oxygen on the basal surface is considered as an ideal HER catalyst [70]. Meanwhile, Jiang et al. [71] successfully synthesized oxygen-functionalized Ti_3_C_2_ MXene (Ti_3_C_2_O_x_) as an HER electrocatalyst in acidic medium. First, dense Ti_3_AlC_2_ powder was etched in HF aqueous solution to prepare layered Ti_3_C_2_T_x_ MXene. Then, the obtained E-Ti_3_C_2_T_x_ was dispersed in 10 wt% KOH aqueous solution to convert the F-terminal group into an OH group to form E-Ti_3_C_2_(OH)_x_. Finally, E-Ti_3_C_2_(OH)_x_ was subjected to an Ar atmosphere at 450 °C for the dehydration reaction to be carried out to obtain oxygen-functionalized Ti_3_C_2_O_x_ MXene, as shown in Figure 7c. Figure 7d demonstrates the SEM image of the laminated Ti_3_C_2_T_x_ MXene formed from the dense Ti_3_AlC_2_ powder obtained via etching in HF aqueous solution. The final product, E-Ti_3_C_2_O_x_, exhibited the best HER catalytic activity compared to E-Ti_3_C_2_T_x_, E-Ti_3_C_2_T_x_-450, and E-Ti_3_C_2_(OH)_x_, as shown in Figure 7e. Due to the presence of highly active O-sites on the Ti_3_C_2_T_x_ MXene substrate, the obtained Ti_3_C_2_O_x_ showed significantly enhanced electrocatalytic activity in an acidic HER, with an overpotential of 190 mV at 10 mA cm^−2^, corresponding to a Tafel slope of 60.7 mV dec^−1^ [48]. Therefore, this surface functionalization approach is considered to be an effective way to improve the electrocatalytic performance of MXenes.

**Figure 8:**
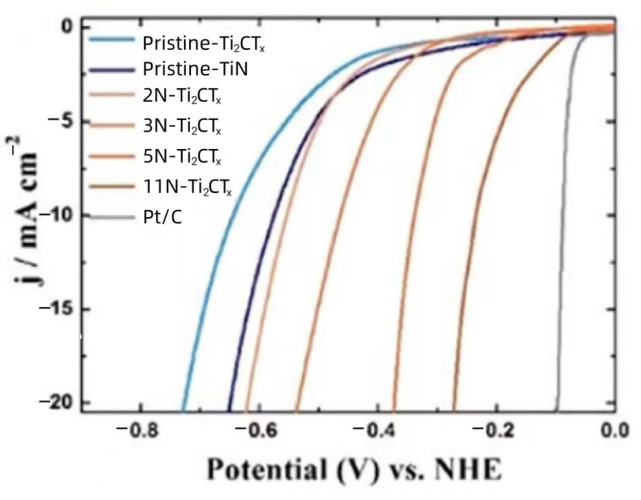
Electrocatalysts were tested using linear sweep voltammetry (LSV curves) at a scan rate of 10 mV s^−1^ without iR correction [72].

In MXene studies, the most common targets were Ti_2_CT_x_ and Ti_3_C_2_T_x_. However, as previously mentioned, Ti_2_CT_x_ had lower catalytic activity for the HER compared to Mo_2_CT_x_, required a higher overpotential (~600 mV), and was less stable in acidic media [67]. Therefore, Ti_2_CT_x_ was considered unsuitable as an HER catalyst. Yoon et al. reported a way for converting non-electrocatalytic Ti_2_CT_x_ generated by using HF etching into an active electrocatalyst by nitriding Ti_2_CT_x_ nanosheets with NaNH_2_ to overcome the low activity of Ti_2_CT_x_ [72]. To achieve the purpose of controlling the level of nitriding, they mixed different doses of NaNH_2_ (20 mg, 40 mg, 80 mg, and 160 mg) with 200 mg of synthesized Ti_2_CT_x_ in a stainless steel autoclave. Depending on the amount of NaNH_2_, the degrees of nitrogenation of the products were 2 at% (2N-Ti_2_CT_x_), 3 at% (3N-Ti_2_CT_x_), 5 at% (5N-Ti_2_CT_x_), and 11 at% (11N-Ti_2_CT_x_). Due to the presence of NaNH_2_, Ti-N_x_ can be considered as an effective HER electrocatalytic active site on the surface of Ti_2_CT_x_ after nitridation treatment [48]. Na and N atoms during the nitriding process dramatically increase the interlayer distance of unstable Ti_2_CT_x_, thus increasing the electrochemical surface area and realizing a productive HER catalyst under acidic conditions. The 11N-Ti_2_CT_x_ catalyst with the highest degree of nitridation has the maximum catalytic activity with a minimal overpotential in 0.5 M H_2_SO_4_ solution, as shown by the LSV curves in Figure 8.

**Figure 9:**
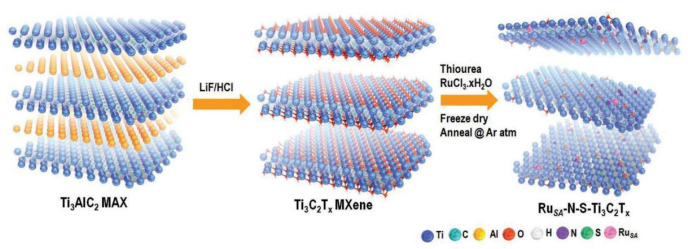
The synthetic process for the catalyst RuSA-N-S-Ti_3_C_2_T_x_ is shown schematically [75].

Some “M” atoms are inevitably etched away during MXene production utilizing fluorine-based aqueous etching, resulting in the development of numerous “M” mono-vacancies or clusters of vacancies, as previously described [73]. Gogotsi et al. demonstrated a positive correlation between the number of vacancy defects in MXene and the concentration of the HF etchant used [74]. In their study, the presence of controllable vacancy defects led to the deformation of the edge lattice, resulting in more electrochemically active sites. Furthermore, because of the extreme instability and reducibility of the “M”-metal vacancy defects in the stripped MXene nanosheets, the addition of metal salts to fill the vacancies can be directly reduced to individual metal atoms. Therefore, the possibility exists to design various single-atom catalysts using MXenes as conducting substrates. In addition, heteroatom doping is an excellent supplemental approach for increasing the loading and adhesion of stabilized single atoms. For instance, Ramalingam et al. created a single-atom catalyst using RuSA-N-S-Ti_3_C_2_T_x_, an N, S co-doped MXene foam with uniformly dispersed Russian metal ions. This catalyst demonstrated outstanding efficiency. As seen in Figure 9, freshly obtained Ti_3_C_2_T_x_, RuCl_3_-xH_2_O, and thiourea were combined, lyophilized, and annealed to yield RuSA-N-S-Ti_3_C_2_T_x_. The catalyst displayed good electrocatalytic efficacy, with an overpotential decrease of 76 mV. In addition, the LSV curve of RuSA-N-S-Ti_3_C_2_T_x_ did not fall on the baseline at zero overpotential, which may be attributed to the noble metal Ru’s underpotential hydrogen adsorption effect and the capacitive impact of the Ti_3_C_2_T_x_ MXene carrier nanocarbon [48]. By N and S co-doping the N and S heteroatoms in Ti_3_C_2_ MXenes, strong RueN and RueS bonds can be formed to immobilize isolated Ru monoatoms and optimize the hydrogen adsorption capacity on the Ru sites [75].

Furthermore, the construction of MXene hybrids is a common approach to enhance HER activity, where other cocatalysts with HER activity, such as transition metal carbides, selenides, and sulfides, are used. A straightforward hydrothermal synthesis of 3D MoSe_2_/MXene hybrid nanoflowers with a low starting potential (61 mV/RHE) was described by Huang et al. as a way to find a novel, advanced electrocatalyst for acid HERs [76]. By introducing Ti_3_C_2_T_x_ with high electrical conductivity as a substrate for MoSe_2_ growth, charge/ion transport was significantly enhanced and the number of active sites was increased. Additionally, the incorporation of the pyridine N dopant carbon to create ternary composite structures is a productive way to maintain the structural integrity of MXene-based materials. For instance, using the in situ polymerization of dopamine and Mo precursors on the Ti_3_C_2_T_x_ MXene surface, Lee’s team was able to successfully create Mo_2_C/Ti_3_C_2_T_x_@NC for an acidic HER [77].

#### 3.1.2. Electrocatalysts for HER in Neutral or Near-Neutral Solutions

To date, most HER studies have been conducted in strongly acidic electrolytes, but there are several drawbacks to HER hydrogen production at very low pH conditions, including chemical corrosion, electrochemical corrosion, and the need for expensive anion/cation exchange membranes. Therefore, neutral/near-neutral HER systems are used as a more sustainable approach to address these issues [78]. H_2_O molecules and dissociated H_3_O^+^ ions are common HER reactants because of the decreased concentration of H_3_O^+^ ions in neutral or nearly neutral solutions compared to strongly acidic electrolytes. In neutral or very neutral electrolytes, the Volmer reaction of H_3_O^+^ ions and H_2_O molecules occurs concurrently; hence, the HER in these fluids follows a more difficult two-stage reduction process [79]. H_3_O^+^ ions are the primary reactants in the first phase, which happens close to the equilibrium potential, in neutral or nearly neutral solutions. The low concentration of H_3_O^+^ ions in a neutral electrolyte is nonetheless sufficient to support the reaction because of the comparatively sluggish reaction rate in this phase. The pH difference between the electrode surface and the native electrolyte caused by the rapid depletion of adjacent H_3_O^+^ ions as the cathodic overpotential rises causes the HER process to switch to diffusion control. This complex two-stage reduction process inevitably leads to slow kinetics inherent in neutral HER, making the search for efficient catalysts a top priority.

So far, neutral electrolytes’ typical Pt-based noble-metal electrocatalysts have received comparatively little research. Zhang et al. prepared Mo_2_TiC_2_T_x_-Pt_SA_ catalysts using in situ electrochemical stripping and atomic capture [17]. Mo vacancies were produced on the surfaces of MXene nanosheets concurrent with electrochemical stripping, and Pt atoms were dispersed from the Pt foil into the electrolyte. The Mo vacancies of Mo_2_TiC_2_T_x_ MXene could firmly immobilize individual Pt atoms, thus exhibiting excellent HER catalytic activity similar to that of Pt/C in neutral solution (0.5 M PBS) at 61 mV/RHE to reach 10 mA cm^−2^. By calculating the density-of-state projections of Mo_2_TiC_2_O_2_ and Mo_2_TiC_2_O_2_-Pt_SA_, it is discovered that the Pt d-orbitals primarily contribute to the density-of-state enhancement of Mo_2_TiC_2_O_2_-Pt_SA_ near the Fermi energy level. This finding suggests that single Pt atoms are able to increase the d-electron predominance near the Fermi energy level and boost the catalytic activity effectively. The single Pt atomic site in Mo_2_TiC_2_O_2_-Pt_SA_ is an excellent active site for hydrogen generation with an adsorption energy of 0.08 eV, which is even lower than that of Pt (−0.10 eV), according to the computed free energy of hydrogen adsorption (ΔG_H*_).

#### 3.1.3. Electrocatalysts for HER in Alkaline Solutions

In industrial electrolyzers, the use of alkaline electrolytes is preferred in order to achieve overall water decomposition as strong alkaline electrolytes (e.g., 20–30% KOH aqueous solutions) help produce high-purity hydrogen [80]. However, there are still many problems associated with strongly alkaline electrolytes such as the electrocatalyst corroding chemically and electrochemically. Therefore, in strongly alkaline electrolytes, stable and effective electrocatalysts are essential. Qiu’s team synthesized mesoporous NiFe-LDH nanosheets in alkaline electrolytes and combined kinetically favorable 3D MXene frameworks (NiFeLDH/MXene/NF) on Ni foams for the HER and OER [81]. They prepared NiFe-LDH/MXene/NF by using electrodeposition wherein the NF was pre-coated with Ti_3_C_2_ MXene as the working electrode. The obtained layered 3D MXene electrode could improve the charge-transfer kinetics and significantly promote the adsorption/activation of H_2_O molecules on NiFe-LDH nanosheets, leading to high-current-density water cleavage in 1.0 M KOH solution. Additionally, the self-supported electrode characteristic reduces intrinsic activity and conductivity degradation brought on by the use of non-conducting binders, such as Nafion, which block active sites, prevent ion diffusion, and raise electron transport resistance [82].

**Figure 10:**
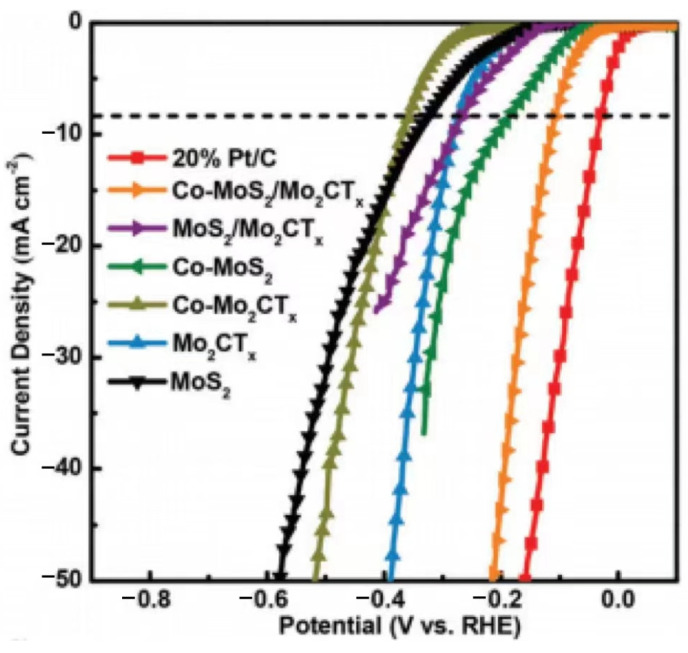
Different materials’ polarization curves when scanned at a rate of 5 mV per second in 1 M KOH (The dashed line shows the theoretical properties of the electrocatalyst in the polarization curve comparison diagram) [84].

MoS_2_, a typical 2D material, is thought to be a promising alkaline HER catalyst. In alkaline solutions, MoS_2_ and MXene composites have a sizable amount of HER catalytic activity [83]. MoS_2_ hybrid materials co-doped with Mo_2_CT_x_ MXene were created by Fan et al. to enhance HER performance in alkaline electrolytes [84]. They prepared Co-MoS_2_/Mo_2_CT_x_, which was produced through wet impregnation using (NH_4_)_2_MoS_4_ and Co(NO_3_)_2_ in the presence of freshly obtained Mo_2_CT_x_ and vacuum dried and annealed under an Ar atmosphere. The LSV curve in Figure 10 shows that Co-MoS_2_/Mo_2_CT_x_ has a small HER overpotential (112 mV). Typically, because of van der Waals forces, MoS_2_ tends to develop parallel to the carrier, creating a composite of 2D materials. Additionally, the MoS_2_ layer’s borders are HER-active sites with high surface energies. MoS_2′_s capacity to grow vertically on a support is therefore advantageous to the stability and activity of hybridized materials. The first vertical development of graphene-modified MoS_2_ hybridized HER catalysts (rGO-MoS_2_/Acc-TiO_2_/C) on MXene-derived accordion-like TiO_2_/C carriers was accomplished by Li et al. The low oxygen resistance of MXenes was eliminated by turning them into TiO_2_/C while keeping their distinctive morphological features. The combination of rGO-MoS_2_/Acc-TiO_2_/C and vertically grown MoS_2_ had a synergistic effect that improved the performance of the alkaline HER electrocatalyst [85]. MXenes and MXene-based materials have been widely studied so far, using a variety of techniques, as HER catalysts in various types of electrolytes, and progress has been achieved in substituting noble-metal catalysts. Table 2 summarizes the studies on different MXene-derived HER catalysts. The electrocatalytic activity and selectivity of MXenes and conventional composite HER catalysts for various typical processes are also shown.

#### 3.1.4. MXene Materials for Hydrogen Storage Applications

At present, MXene materials have been widely used as catalysts in the hydrogen storage field. Among their uses, a common application has been as a catalyst for MgH_2_. By adding Ti_2_C to MgH_2_, Li et al. found that 2DTi_2_C MXene had a good catalytic effect on the dehydrogenation of MgH_2_ [92]. Compared with pure MgH_2_, the initial dehydrogenation temperature, apparent activation energy (Ea), and total enthalpy change (ΔH) of MgH_2_ with 5% (mass fraction) Ti_2_C decreased by 37 °C, 36.5%, and 11%, respectively. Similarly, MgH_2_ containing 5% (mass fraction) Ti_3_C_2_ had a lower initial dehydrogenation temperature (93 °C) compared to the original sample and began to absorb hydrogen at room temperature, inhaling 6.2% (mass fraction) hydrogen in just 30 s at 150 °C [93]. This highly catalytically active characteristic of Ti_3_C_2_ is mainly attributed to its unique layered structure and in situ formation of metal Ti. In addition to MXenes composed of Ti-C, some other MXene materials have been proven to have a certain effect in terms of improving hydrogen desorption thermodynamics. Researchers successfully synthesized a novel solid-solution MXene (Ti_0.5_V_0.5_)_3_C_2_ by stripping the solid-solution MAX phase (Ti_0.5_V_0.5_)_3_AlC_2_. Experimental results show that adding a small amount of (Ti_0.5_V_0.5_)_3_C_2_ can significantly reduce the operating temperature of Mg hydrogen uptake and release and improve the reversibility of hydrogen storage. Among hydrogen storage materials, complex hydrides have high weight and volume hydrogen capacity, making them promising materials that can meet the needs of practical applications. NaAlH_4_ is considered to be the best hydrogen storage material for practical applications among the known light-metal coordination aluminum hydrides. Several feasible strategies have been proposed to adjust the thermodynamic and kinetic properties of hydrogen storage in NaAlH_4_ such as doping catalysts, creating active composites, and making nanorefinements. A large number of studies have proven that catalyst doping is one of the most effective methods to solve the problem of dehydrogenation and refill kinetics of NaAlH_4_. Bogdanovic et al. proposed a novel reversible hydrogen storage system based on a catalytic reaction, that is, adding a small amount of titanium compounds to alkali aluminum hydride to achieve bidirectional catalytic acceleration [94]. In addition to MG-based and Na-based materials, Li-based materials are also potential hydrogen storage materials. Zang et al. synthesized a LiBH_4_@Ti_3_C_2_ hybrid by introducing LiBH_4_ into the layered structure of Ti_3_C_2_ using a simple impregnation method [95]. The initial desorption temperature of LiBH_4_@Ti_3_C_2_ hybrid was reduced to 172.6 °C, which was significantly lower than that of pure LiBH_4_. At the same time, the sample showed good dissociation kinetics, with 9.6% (mass fraction) of hydrogen removed from LiBH_4_ at 380 °C for 1 h, and its activation energy decreased by about 50% compared with pure LiBH_4_. From the perspective of the mechanism, the improvement in the hydrogen storage performance of nano-LiBH_4_ occurred due to the synergistic effect of the unique Ti_3_C_2_-layered structure of nano-LiBH_4_ and the instability activity caused by titanium-containing defect sites. Similarly, Fan et al. reached a similar conclusion when using Ti_3_C_2_ MXene to improve the hydrogen storage performance of LiBH_4_ [96].

### 3.2. Electrocatalysts Based on MXene for the OER

The OER is essential to many crucial processes for converting and storing renewable energy, including metal–air batteries and electrochemical water breakdown. However, the need for high-performance catalysts is urgent given the sluggish kinetics and significant overpotential of the OER. Although noble-metal oxides (e.g., RuO_2_ and IrO_2_) are high-performance catalysts for OER, their high cost and scarcity severely limit their wider application. Consequently, people have been looking for precious-metal-based alternative materials with abundant reserves and high activity.

**Figure 11:**
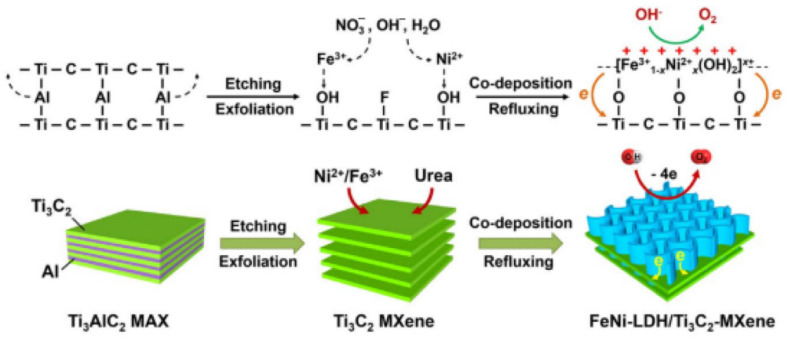
The process of preparing layered FeNi-LDH/Ti_3_C_2_T_x_ nanohybrids through the co-precipitation of Ni^2+^ and Fe^3+^ [99].

MXene-based materials have drawn a lot of interest because of their potential applications in the OER. For example, Yu et al. prepared layered FeNi-LDH/Ti_3_C_2_T_x_ nanohybrids by co-precipitating Ni^2+^ and Fe^3+^ in Ti_3_C_2_T_x_ and urea, as shown in Figure 11 [13]. With a current density of 10 mA/cm^2^ and a 298 mV overpotential, the synthesized hybrids displayed good OER activity. They also displayed a low Tafel slope of 43 mV/dec. This success can be due to Ti_3_C_2_T_x_ and FeNi-LDH’s significant charge transfer, robust interfacial contact, and electronic coupling. This interaction and coupling significantly accelerated the redox process of converting FeNi-LDH to OER while also improving conductivity and stability. Through the layer-by-layer self-assembly of graphitic carbon nitride (g-C_3_N_4_) with titanium carbide (Ti_3_C_2_), Ma et al. created freestanding flexible thin films [97]. In actuality, the catalysts demonstrated a negligible Tafel slope of 74.6 mV/dec in 0.1 mol/L KOH solution and only required a modest overpotential of 420 mV to reach a current density of 10 mA/cm^2^. The resulting hierarchical porous films were extremely hydrophilic and had good OER activity. The catalyst’s Ti-Nx motif, which serves as the active site, was credited with its outstanding performance. By using XPS and near-edge X-ray-absorption fine-structure spectroscopy, this theory was confirmed. Tang et al. constructed S-NiFe_2_O_4_@Ti_3_C_2_@NF hybrids and investigated their OER performance [98]. The hybrid materials showed an overpotential of 270 mV, a current density of 20 mA/cm^2^, and a small Tafel slope of 46.8 mV/dec in 1 mol/L KOH solution.

To improve OER performance, metal–organic skeletons (MOFs) and MOF derivatives have been effectively hybridized with MXene nanosheets. Zhao et al., for example, used a mutual-diffusion-reaction-assisted approach to create MXene/MOF hybrids (Ti_3_C_2_T_x_-CoBDC) [100]. The hybrids required a low overpotential of 410 mV in 0.1 mol/L KOH solution to produce a current density of 10 mA/cm^2^ and had a low Tafel slope of 48.2 mV/min. The impressive OER performance was possible due to the clearly defined interface between the codc layer and the Ti_3_C_2_T_x_ nanosheets, which enabled rapid charging and ion transfer. Metallic Ti_3_C_2_T_x_ nanosheets were added, which enhanced charge and ion transport in addition to preventing the porous codc layer from aggregating. In another job, CoNi-ZIF-67@Ti_3_C_2_T_x_ was prepared using a simple co-precipitation reaction [101]. Because of the presence of Ti_3_C_2_T_x_, the average Co/Ni element oxidation increased and the CoNi-ZIF-67 particle size decreased, which led to the excellent OER performance of the catalysts. The CoNi-ZIF-67@Ti_3_C_2_T_x_ mixture had a starting potential pair RHE that was low, at 275 mV, and the Tafel slope was 65.1 mV/dec. Zou et al. prepared a novel NiCoS/Ti_3_C_2_T_x_ hybrid using a MOF-based method [102]. The hybrid has an overpotential of 365 mV and a Tafel slope of 58.2 mV/dec at 10 mA/cm^2^ and shows high stability.

**Figure 12:**
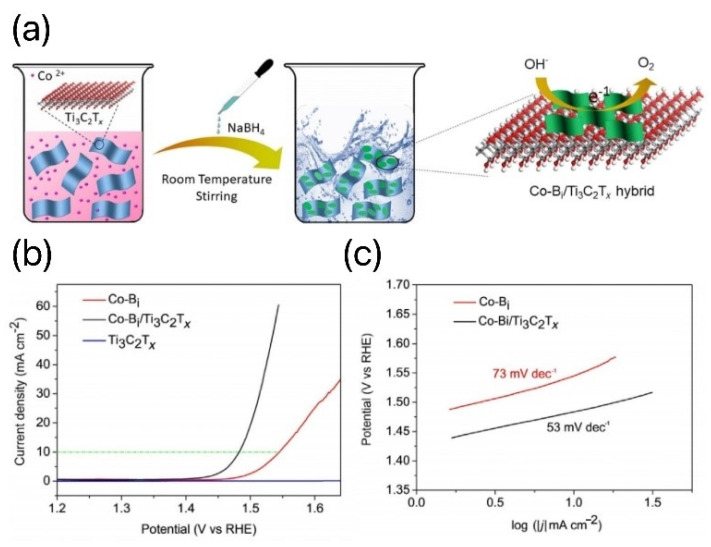
(**a**) Preparation process for the hierarchical Co-Bi/Ti_3_C_2_T_x_ hybrid. (**b**) Polarization curves. (**c**) Tafel plots of Co-Bi/Ti_3_C_2_T_x_ hybrid and Co-Bi nanosheets [103].

Many MXene-based hybrids have intriguing OER applications. For instance, Liu et al. used a rapid chemical reaction at ambient temperature to create a one-of-a-kind layered cobalt borate/Ti_3_C_2_T_x_ MXene (Co-Bi/Ti_3_C_2_T_x_) [103]. Metallic Ti_3_C_2_T_x_ nanosheets not only improved the material’s electron transport capabilities but also hampered the aggregation of Co-Bi nanosheets. Strong charge-transfer capacities were provided by the association of Ti_3_C_2_T_x_ and Co-Bi nanosheets, which significantly increased the electrostatic attraction of additional anionic intermediates, resulting in a rapid redox process. As a result, the synthesized mixtures performed exceptionally well in terms of the OER, with overpotentials as low as 250 mV (10 mA/cm^2^) and a modest Tafel slope of 53 mV/dec, as shown in Figure 12. Table 3 gives lists the results of conventional composite catalysts for MXenes and MXene-derived OERs and summarizes the electrocatalytic activity and selectivity of various common processes.

### 3.3. MXene-Based Electrocatalysts for the ORR

**Figure 13:**
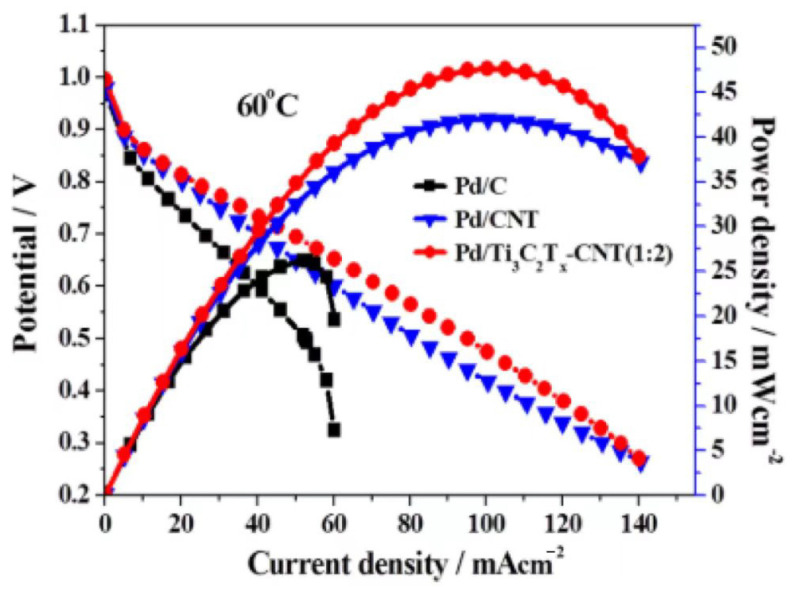
Polarization curves for MEA 2 (anode: Pt/C, cathode) [112].

The reduction of cathodic oxygen is an important process in fuel-cell power generation, but this process is limited by slow kinetics [2]. The performance of pristine Ti_3_C_2_T_x_ in terms of the ORR is barely up to the mark, but MXenes can be used in combination with other ORR-active materials for synergistic enhancement. Chenxi Xu et al. showed that the performance of electrocatalysts could be improved using a hybridized catalytic substrate consisting of MXene (Ti_3_C_2_T_x_) and carbon nanotubes [112]. Compared with the Pd/C catalyst, this hybridized catalyst had higher durability and ORR activity. For the ORR reaction, the mass specific and specific activities of the Pd/Ti_3_C_2_T_x_-CNT catalyst were 4.4 and 3.3 times higher than those of the Pd/C catalyst, respectively. In particular, the Pd/Ti_3_C_2_T_x_-CNT (1:2) catalyst had a higher cell voltage and a lower activation overpotential loss and higher ORR catalytic activity compared to the Pd/C catalyst. This improved catalyst performance may be due to stronger metal carrier contacts and/or faster interfacial oxygen kinetics. The peak power densities of Pd/C, Pd/CNT, and Pd/Ti_3_C_2_T_x_-CNT(1:1) catalysts were 26.2 mW cm^−2^, 42 mW cm^−2^, and 48 mW cm^−2^, respectively, at 60 °C. These findings suggest that Pd/Ti_3_C_2_T_x_-CNT(1:2) can be used as a superior catalyst, as shown in Figure 13. In addition, a FePc/Ti_3_C_2_T_x_ hybrid catalyst prepared by loading iron phthalocyanine on Ti_3_C_2_T_x_ MXene can also significantly improve the catalytic activity. The interaction of Ti_3_C_2_T_x_ MXene surface phases with FeN_4_ induces significant three-dimensional electron delocalization and spin-state jumps of Fe(II) ions, which facilitate reactive-oxygen-species adsorption and reduction, with catalytic activity being four times higher than that of unloaded FePc.

Since MXenes have good oxygen adsorption sites and can synergize with electrocatalytically active compounds, they also operate as bifunctional electrocatalysts for the electrochemical OER and ORR. First-principles calculations were used to estimate the catalytic activity of F-, OH-, and O-terminated Ti_2_C and non-terminated Ti_2_C, which suggests that functional group-engineered MXenes may be very active for both the OER and ORR [113]. Furthermore, the addition of other non-precious metals to the MXene surface (e.g., Fe/Co/Ni) has been reported, enabling the effective synthesis of bifunctional MXene-based catalysts [114]. These modified Ni_1_/Ni_2_ and Fe_1_/Ni_2_ MXene-based diatomic catalysts (DACs) outperformed the well-known low-overpotential monofunctional catalysts (Pt/C and IrO_2_/C) in terms of ORR/OER activities according to experimental findings and theoretical calculations [115]. In addition, a surfactant-free method was used to successfully create bifunctional catalysts based on 3D MXenes and nitrogen-doped cobalt selenide nanocrystals (N-CoSe_2_/3D Ti_3_C_2_T_x_). Since Ti_3_C_2_T_x_ and CoSe_2_ made strong contact, a considerable number of electrons were transferred as a result, and the catalyst’s electrocatalytic performance was astounding [116]. The OER and ORR routes were reported to have all reaction intermediates that energetically bind to Co sites exposed on the surface of N-CoSe_2_/3D Ti_3_C_2_T_x_, proving that they were the intrinsic active sites for both processes. In conclusion, the design of active sites and the modulation of the catalytic atomic environment are crucial in optimizing the electrochemical behavior of MXenes, especially when constructing MXene-based electrocatalysts for the ORR or OER.

Table 4 summarizes the progress of research on MXenes and MXene-derived ORR catalysts and describes the electrocatalytic activity and selectivity of MXene and conventional composite ORR catalysts in a variety of typical processes.

### 3.4. MXene-Based Electrocatalysts for the NRR

**Figure 14:**
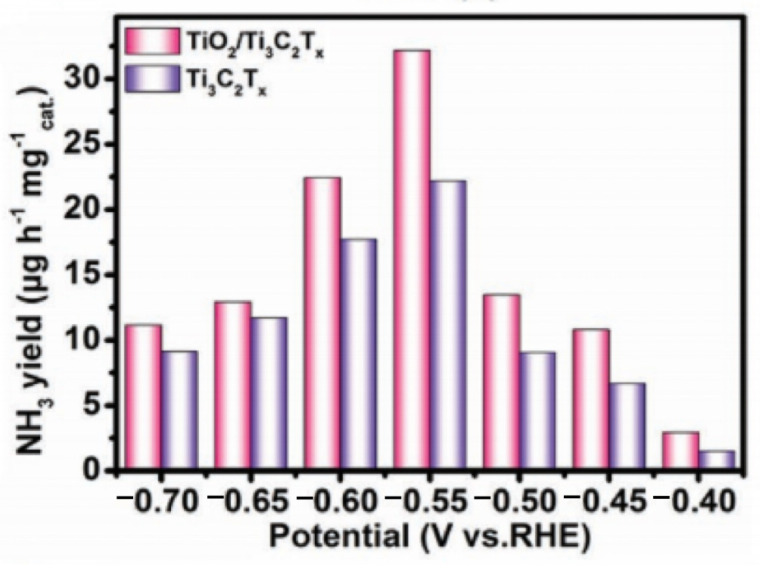
The yield rate of NH_3_ production [125].

Because of the scientific, socioeconomic, and environmental importance of NH_3_, much emphasis has been paid to research on the electrocatalytic conversion of N_2_ to NH_3_ in environmental settings. However, the development of NRR is severely hindered by the lack of effective electrocatalysts [124]. Recently, the potential of MXenes as constituting an effective platform for nitrogen reduction has been investigated using DFT calculations, and they are considered to be promising N_2_ trapping materials with better N_2_ trapping ability than thermodynamically assumed CO_2_ and H_2_O. When nitrogen is chemisorbed on MXene nanosheets, it is activated simultaneously. The overpotentials of V_3_C_2_ and Nb_3_C_2_ can be reduced to 0.64 V and 0.90 V. Since most of the NRR active sites in MXenes are marginal-transition-metal M, researchers can further improve their NRR activity by exposing more active sites or reducing the selectivity of competitive HER reactions. To catalyze the NRR reaction, Luo et al. designed a Ti_3_C_2_T_x_/FeOOH composite [15]. Their results showed that the NRR active site of the material was Ti at −0.1 V vs. RHE, and the maximum yield of NH_3_ was 4.72 μg h^−1^ cm^−2^, while the Faraday efficiency was 5.78% at −0.2 V vs. RHE. The performance of the material was maintained at 72.7% after durability tests with six timed runs. This was due to the low HER selectivity of FeOOH, which modifies the high HER selectivity of the MXene surface-functional groups, thus reducing the HER competition in the overall NRR reaction. Meanwhile, the Ti_3_C_2_T_x_ grown on FeOOH has a small size and high dispersion due to its vertical alignment, which exposes more marginal Ti active sites and thus improves the catalytic activity for NRR. In addition, through the in situ derivatization of transition metals on MXenes, higher active sites can be generated, which can further improve the NRR catalytic performance of MXene materials. Fang et al. prepared layered TiO_2_/Ti_3_C_2_T_x_ for electrocatalytic NRRs by using Ti atoms for the in situ growth of TiO_2_ on Ti_3_C_2_T_x_ [125]. As shown in Figure 14, the oxygen vacancies in TiO_2_ are the main active sites, with an NH_3_ yield of 32.17 mg h^−1^ mg^−1^_cat_ (−0.55 V vs. RHE, 0.1 M HCl) and a Faraday efficiency of 16.07% (−0.45 V vs. RHE). In addition, the TiO_2_/Ti_3_C_2_T_x_ catalyst was capable of achieving 50 h of continuous NRR electrocatalysis. The superior NRR catalytic performance was attributed to the fact that the composites formed a large number of oxygen vacancies during catalyst preparation, which increased the number of active sites. Meanwhile, the combination of the two different materials not only produced a synergistic effect, which favored the charge transfer and electron injection of the oxygen vacancies, but also increased the bond length of nrn. Furthermore, recent research has shown that the primary NRR-active sites can be thought of as the oxygen vacancies of TiO_2_ nanoparticles (TiO_2_/Ti_3_C_2_T_x_) produced in situ on Ti_3_C_2_T_x_ nanosheets. The Faraday efficiencies of the prepared electrocatalysts were 2.26 times higher than those of Ti_3_C_2_T_x_, and DFT simulations revealed that the reaction energy barriers for TiO_2_/Ti_3_C_2_T_x_ in NRR were the lowest. The highly conductive MXenes promoted TiO_2_ synthesis and prevented TiO_2_ nanoparticle self-aggregation in addition to facilitating electron transfer in the NH_3_-catalyzed process and enhancing NRR performance. The designing of MXene-based electrocatalytic nitrogen fixation and NH_3_ generation is made possible by these investigations. Various MXenes have been investigated for their potential as electrochemical NRRs, as shown in Table 5.

### 3.5. MXene-Based Electrocatalysts for the CO_2_RR

**Figure 15:**
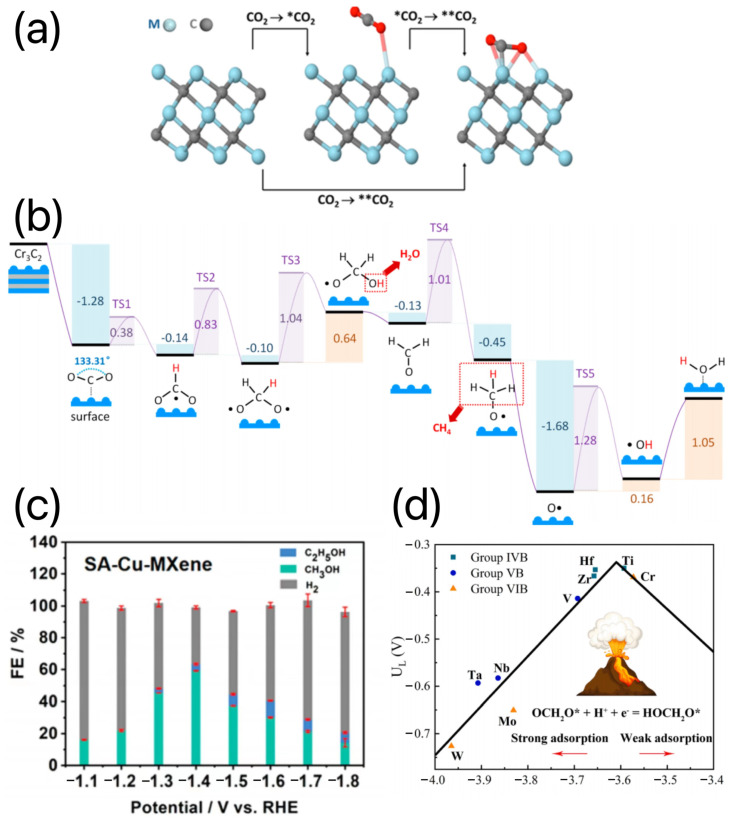
(**a**) The metal surfaces of MXenes adsorb CO_2_ through physical adsorption, chemisorption, and interaction. “M” and “C” refer to octahedral coordination transition metals and carbon atoms, respectively; The * and ** symbols denote substances that are physically and chemically absorbed, respectively. (**b**) O^−^- and OH-functionalized Cr_3_C_2_ and Mo_3_C_2_ MXenes can significantly reduce the energy barrier of CO_2_ conversion to CH_4_ [130]. (**c**) FEs of SA-Cu-Mxene [131]. (**d**) The CO_2_RR volcano plot of Mo_3_C_2_ and TM-substituted bimetal MXenes. OCHO* represents the possible products of hydrogenation [132].

Owing to the substantial carbon dioxide (CO_2_) emissions brought on by burning fossil fuels, there has been an increase in global climate change. Using electrocatalytic CO_2_RR is thought to be a potential method for converting CO_2_ into useful materials and fuels [129]. MXenes and MXene-based materials are characterized by high electrical conductivity and tunable structures and are therefore considered as potential CO_2_RR electrocatalysts. As an example, Cr_3_C_2_ and Mo_3_C_2_ MXenes have been proposed to be used for the selective conversion of CO_2_ to CH_4_. The metal-terminated surfaces of MXenes adsorb CO_2_, first through physical adsorption and then via chemisorption/interaction, as shown in Figure 15a. In particular, the O- and OH-functionalized Cr_3_C_2_ and Mo_3_C_2_ MXenes can significantly lower the energy barrier for the conversion of CO_2_ to CH_4_, as shown in Figure 15b [130]. Zhao et al. demonstrated the selective etching of the Al layer in a four-element MAX (A = Al and Cu) to obtain accordion-shaped Cu-Ti_3_C_2_Cl_x_ wherein copper atoms were predominantly fixed on the MXene surface in the form of Cu-O coordination [131]. Cu-Ti_3_C_2_Cl_x_ exhibited high selectivity for methanol in the electrocatalytic CO_2_ reduction process, with an optimal copper content of 2 wt%. The Faradaic efficiency reached 59.1% at −1.4 V, with a charge-transfer resistance (C_dI_) of 9.7 mF cm^−2^, and stable current density, as shown in Figure 15c. Furthermore, the Faradaic efficiency could be maintained at around 58%, and after a stability test of 30 h, the material was found to be resistant to oxidation. This was because dispersed copper atoms can inhibit the C-C coupling of C1 intermediates such as *CO, reducing the formation of C^2+^ products like ethanol. Additionally, the electronic structure of single-atom copper is unsaturated. Bharath et al. designed a Pd50-Ru50/Ti_3_C_2_T_x_ catalyst for CO_2_ electrocatalytic hydrogenation that exhibited high selectivity towards methanol products at low temperatures with a methanol yield of approximately 76% [131]. At a catalyst loading of 20 mg, the maximum conversion rate of carbon dioxide was about 78%, and the total turnover number (TON) reached 2932. This was attributed to the loading of Pd_50_-Ru_50_ on Ti_3_C_2_T_x_, which effectively prevents the aggregation of Pd_50_-Ru_50_, while Pd_50_-Ru_50_ prevents the aggregation of MXene. The mesoporous structure formed by the combination of Pd50-Ru50 and Ti_3_C_2_T_x_ has a high specific surface area (120 m^2^ g^−1^), exposing more active sites. It was also found that extending the reaction time can lead to formic acid formation, promoting methanol production. In contrast to previous studies on single-atom metal-doped MXene, Li et al. investigated the electrocatalytic reduction of CO_2_ by substituting Mo in Mo_3_C_2_ with Ti, Zr, Hf, V, Nb, Ta, Cr, and W [132]. It was found that the complete substitution of the interlayer resulted in the best catalytic performance for CO_2_ reduction, with the occurrence of highly selective methane synthesis and the suppression of competing reactions with water reduction. Among them, Mo_2_TiC_2_ exhibited the best electrocatalytic performance for CO_2_RR, lowering the limiting potential of MXene from −0.651 to −0.350 V, as shown in Figure 15d. This improvement can be attributed not only to the disruption of linear scaling relationships between intermediate adsorption energies caused by transition metal doping, which severely hinders catalytic efficiency, but also to the presence of Ti, which provides Mo with stronger localized lone-pair states. Table 6 describes the electrocatalytic activity and selectivity of MXene and conventional CO_2_RR catalysts in a variety of typical processes.

These studies laid the foundation for the utilization of MXenes for CO_2_ capture and demonstrated their potential as novel CO_2_RR catalysts. In conclusion, through effective MXenes nanostructure design and rational electronic structure modulation, the specific surface areas of MXenes can be significantly increased, charge or ion transfer can be accelerated, internal activity can be enhanced, more active sites can be provided, and the reaction kinetics can be promoted, which can significantly improve the catalytic performance and the prospects of the electrocatalytic applications of MXenes.

As mentioned earlier, it is important for MXene electrocatalysis to elucidate the actual catalytic process, the main role of the catalytic active site, and the underlying mechanism of catalysis. In addition, the future development of MXene electrocatalysis is mainly focused on the following aspects:

1. Control of surface properties: This occurs through crystal surface control, morphology and size control, defect control, and other strategies to design the catalyst to improve its surface properties. These methods can adjust the surface structure and chemical composition of the catalyst and improve the catalytic activity.
2. Polymetallic MXene: Researchers can synthesize MXene materials composed of multiple metallic elements, using the synergistic and geometric effects of polymetallic MXene to directly produce directional electron distribution and more active sites, thereby improving electrocatalytic performance.
3. Composite cocatalysis: MXenes can be used as conductive enhancers in composites with strong interfacial coupling and fast charge-transfer kinetics. Therefore, combining MXene with other functional materials to form composite materials can effectively improve the electrochemical properties of composite materials.

These modification methods can improve the catalytic performance of the catalyst and bring new opportunities for the expansion of the MXene family in the future.

## 4. Conclusions

MXenes as newly discovered 2D materials exhibit excellent electrochemical energy conversion potential. In this paper, a systematic review of MXene-based electrocatalytic materials has been presented, including the research progress related to the HER, OER, ORR, NRR and CO_2_RR. MXene-based high-performance catalysts have excellent properties and performance, including high electrical conductivity, adjustable morphology and bandgap structure, large specific surface area, and more. The main preparation methods of MXenes have been firstly introduced, and different methods have their advantages and disadvantages. The research on and application of MXenes in the field of electrocatalysis in the past five years have been highlighted.

Despite the exciting advances in the field of electrocatalysis with MXenes, there are still a number of challenges that need to be addressed. First, MXene materials are poorly stabilized, and the preparation methods are hazardous. They are easily oxidized in air, and the commonly used acid etching process is toxic and dangerous. Therefore, there is an urgent need to address the stability of MXene materials and to explore environmentally friendly and efficient routes for mass-production preparation. Second, the electrocatalytic properties of MXenes are significantly affected by their surface functional groups. In addition, due to the limitation of the production process, relatively few electrocatalytic studies have been conducted for nitride MXenes and other types of MXene materials, which has limited the development of MXene materials with higher electrocatalytic properties. Therefore, it is crucial to improve the preparation process for MXenes and expand the types of MXenes.

In summary, the future development of MXene electrocatalysis focuses on the following aspects: finding safe, environmentally friendly, efficient, and inexpensive preparation methods to obtain large-scale MXene materials with controllable numbers of layers, excellent quality, porosity, and monolithic nature; designing catalysts with good catalytic performance through surface property modulation; developing multimetallic MXene and utilizing synergistic and geometrical effects to improve electrocatalytic performance; and using MXenes as conductive enhancers to improve the electrochemical performance of composites. All of these improvements can enhance the performance of catalysts and will bring new opportunities for the expansion of the MXene family.

At the same time, MXene research on the hydrogen storage properties of materials has contributed to changes in the field of hydrogen storage materials. If applied directly to hydrogen storage, the interaction between hydrogen atoms and hydrogen molecules on the surface of MXene can be utilized in various ways to achieve optimal adsorption. Meanwhile, the functional groups and doped metals on the surface of MXene also play a crucial role in hydrogen adsorption performance. Coupled with their two-dimensional properties, the adsorption capacity of hydrogen atoms varies with different layer spacings. If MXene is used to modify conventional hydrogen storage materials, high-capacity and reversible hydrogen storage can be realized, and significant enhancement in hydrogen storage performance can be achieved, which proves that MXene has considerable advantages in hydrogen storage applications. However, the current theoretical calculations of first principles are mainly for MXene composed of Ti-C elements, and so, theoretical calculations can be attempted for other MXene phases to find out whether there are materials with better performance. Also, the preparation method can be improved, and even other metals can be doped to improve the hydrogen adsorption capacity. In summary, MXene materials have great potential for development in the field of hydrogen storage. Research on them promotes the progress of hydrogen storage technology and provides an ideal choice for future hydrogen storage technology.

In conclusion, since the debut of MXenes in 2011, researchers have been working hard to continuously discover the multifunctionality and prospects of MXenes. In recent years, many innovative and significant advances have been made in MXene-based electrocatalytic research. However, the current research is still in its infancy. It is still challenging to evaluate the catalytic performance of MXene-based catalysts and to elucidate their catalytic mechanisms. In addition, MXene has not been extensively studied, except for Ti_3_C_2_T_x_, which deserves further discussion in the future. We hope that this review can provide a fundamental guide for scientific research engaged in a variety of MXene-assisted electrocatalytic processes and help to deeply understand the challenges and opportunities involved. We also hope that it will inspire more breakthroughs on the fundamental mechanisms, new design approaches, and even scaled-up integrated systems for MXene-assisted multiple electrocatalytic processes. In the future, with further exploration and research, we believe that MXenes will achieve more progress and applications in the field of electrocatalysis.

## Figures and Tables

**Table 1:** Summary of the main reviews related to recent advances in the synthesis of MXene materials and its application in electrocatalysis in the present review.

Title	Focus	Ref.
Progress in the synthesis process and electrocatalytic application of MXene materials	The preparation methods of xylene are reviewed in detail. The research progress in electrocatalysis and the key factors affecting the properties of the materials, such as the functional groups, electrical conductivity, and interface, are summarized. The main challenges and opportunities facing MXene materials in basic research and practical applications as a next generation electrocatalytic platform are highlighted.	This work
Recent advances in noble metal MXene-based catalysts for electrocatalysis	The paper reviewed the strategies for the synthesis of noble-metal MXene-based catalysts, focusing on the application of noble-metal MXene-based catalysts in the field of electrocatalysis and highlighting the strategies for improving the electrocatalytic performance of noble-metal MXene-based catalysts.	[19]
Recent advances in structural engineering of MXene electrocatalysts	Representative advances in MXenes as electrocatalysts for hydrogen evolution reactions were reviewed both experimentally and theoretically.	[20]
2D MXene Nanomaterials as Electrocatalysts for Hydrogen Evolution Reaction (HER): A Review	Recent advances in the synthesis and HER performance of MXene-based electrocatalysts were summarized from both theoretical and experimental perspectives. The advantages of MXene-based catalysts over conventional Pt/C catalysts in terms of HER kinetics, Tafel slopes, overpotentials, and stability in acidic and alkaline electrolytic environments were systematically evaluated.	[21]
A Review on MXene as Promising Support Materials for Oxygen Evolution Reaction Catalysts	The role of MXenes as support materials in improving the performance of OER catalysts was emphasized.	[22]
Recent Advances on MXene-Based Electrocatalysts toward Oxygen Reduction Reaction: A Focused Review	Current research on MXenes for ORR was discussed, focusing on synthesis strategies, ORR activity, and factors responsible for improving electrocatalytic performance. Several strategies for further development of efficient and durable ORR-based MXene catalysts were also presented.	[23]
Recent advances of MXene as promising catalysts for electrochemical nitrogen reduction reaction	Recent advances on MXene-based catalysts for electrochemical N_2_ reduction reactions (NRRs) were emphasized. With respect to providing guidelines for exploring more efficient MXene-based catalysts for NRR, the preparation and surface modification of MXene were discussed. In addition, the shortcomings and challenges of current research were summarized, and future research directions were envisioned.	[24]
Photocatalytic and electrocatalytic reduction of CO_2_ by MXene-based nanomaterials: A review	A comprehensive review of the current findings on the photocatalytic and electrocatalytic reduction of CO_2_ by various MXene-based nanomaterials was presented. The review focused on the (i) photocatalytic reduction of CO_2_ by functionalized Ti_3_C_2_, TiO_2_/Ti_3_C_2_, g-C_3_ N_4_/Ti_3_C_2_, and other/Ti_3_C_2_ catalysts, (ii) electrocatalytic CO_2_ reduction, (iii) CO_2_ reduction associated with photothermal catalysis and hydrogenation, and (iv) the stability of MXene-based photocatalysts.	[25]
Applications of 2D Mxenes for Electrochemical Energy Conversion and Storage	This paper highlighted the preparation methods and special features of MXenes in terms of electrode materials, conductive substrates, surface modification, heteroatom doping, wrinkling, and protective layers against dendrite growth.	[26]
MXenes: Emerging 2D materials for hydrogen storage	In this paper, the application status, challenges, and future prospects of hydrogen storage materials based on MXene were reviewed.	[27]

**Table 2:** Electrochemical performance of MXenes as HER catalysts.

Electrocatalyst	Substrate	Mass Loading [mg cm^−2^]	Overpotential η [mV]	Tafel Slope [mV dec^−1^]	Solution	Ref.
Pt/Ti_3_C_2_T_x_	GCE	0.38	55	65	Acidic	[24]
Pt/3D Ti_3_C_2_	GCE	0.2	27	41	Alkaline	[86]
Pt–SnS_2_	—	—	117	69	Acidic	[87]
Ti_3_C_2_T_x_/Ni_3_S_2_	NF	4.9	72	45	Alkaline	[88]
TiOF_2_/Ti_3_C_2_T_x_	GCE	0.18	197	56.2	Acidic	[89]
Co/Mo_2_CT_x_	GCE	0.1	180	59	Acidic	[90]
Co–MoS_2_/Mo_2_CT_x_	GCE	0.35	112	82	Acidic	[84]
CoMoS	FTO	—	282	—	Neutral	[91]

**Table 3:** Electrochemical performance of MXene OER catalysts.

Electrocatalyst	Substrate	Mass Loading [mg cm^−2^]	Overpotential η [mV]	Tafel Slope [mV dec^−1^]	Solution	Ref.
CoP/Ti_3_C_2_T_x_	CFP	1.5	230	50	Alkaline	[104]
Co^3+^/Ti_2_CT_x_	GCE	0.1	—	132	Alkaline	[105]
Co-LDH@Ti_3_C_2_T_x_	GEC	0.35	330	32	—	[106]
FeCo-LDH/Ti_3_C_2_T_x_	GEC	0.357	268	85	—	[107]
NiFe/Ti_3_C_2_T_x_	—	0.36	260	—	Alkaline	[108]
FeNi_2_Se_4_–NrGO	CFP	—	170	62.1	Alkaline	[109]
Ni_3_Se_2_	Au	—	290	97.1	Alkaline	[110]
NiFe LDH/rGO	Ni foam	—	200/210	40	Alkaline	[111]

**Table 4:** Electrochemical performance of MXene ORR catalysts.

Electrocatalyst	Half-Wave Potential (V)	Mass Loading [mg cm^−2^]	Onset Potential (V vs. RHE)	Tafel Slope [mV dec^−1^]	Solution	Ref.
FeNC/Ti_3_C_2_T_x_	0.814	0.1	−1	30	—	[117]
Fe–N–C/Ti_3_C_2_T_x_	0.84	0.1	−0.92	—	Alkaline	[118]
FeN_x_C/C–F	0.76	0.8	−0.88	—	Acidic	[119]
mNC–Fe_3_O_4_@rGO-2	0.83	0.24	−0.96	—	Alkaline	[120]
MoS_2_–Ti_3_C_2_T_x_/MWCNTs	0.75	—	−0.87	90	Alkaline	[121]
Co–CNTs/Ti_3_C_2_T_x_	0.82	—	—	63	—	[122]
g-C_3_N_4_/Ti_3_C_2_T_x_	0.79	0.4	−0.92	—	Alkaline	[14]
CPANI/Mn_2_O_3_	0.68	0.28	−0.83	—	Alkaline	[123]

**Table 5:** Electrochemical performance of MXene NRR catalysts.

Electrocatalyst	Substrate	Mass Loading [mg cm^−2^]	Potential η [mV]	Electrolyte	Product Yield [μg h^−1^ mg_cat_^−1^]	Faradic Efficiency [%]	Ref.
Ti_3_C_2_T_x_ (T = O, OH)	Carbon cloth	0.8	−0.3	0.01 M HCl	36.9	9.1	[126]
Ti_3_C_2_T_x_	CP	0.2	−0.4	0.01 M HCl	20.4	9.30	[127]
TiO_2_/Ti_3_C_2_T_x_	CP	0.1	−0.55	0.01 M HCl	32.17	8	[125]
Ru/Mo_2_CT_x_	CP	0.3	−0.3	0.5 M K_2_SO_4_	40.57	25.77	[128]
Ru/Ti_3_C_2_T_x_	GCE	1.02	−0.4	0.1 M KOH	38.33	13.13	[125]

**Table 6:** Electrochemical performance of MXene CO_2_RR catalysts.

Electrocatalyst	Substrate	Method(s)	Electrolyte	Faradic Efficiency [%]	Ref.
Pd/NbN	—	Heteroatom doping	0.5 m NaHCO_3_	38.4	[133]
Ti_2_CT_x_	—	Termination engineering	0.1 m KHCO_3_	56.1	[134]
SA-Cu-MXene	GCE	Heteroatom doping	0.1 m KHCO_3_	59.1	[131]
NTC-VTi	CP	Heteroatom doping/defect engineering	Seawater	92	[135]

## Data Availability

Not applicable.
